# Preparation of Black Ceramic Tiles Using Waste Copper Slag and Stainless Steel Slag of Electric Arc Furnace

**DOI:** 10.3390/ma13030776

**Published:** 2020-02-08

**Authors:** Mengke Liu, Guojun Ma, Xiang Zhang, Junjie Liu, Qiang Wang

**Affiliations:** 1 The State Key Laboratory of Refractories and Metallurgy, Wuhan University of Science and Technology, Wuhan 430081, China; liumengke@wust.edu.cn (M.L.); zx91@wust.edu.cn (X.Z.); qwe1023215227@163.com (J.L.); wangqiang58@wust.edu.cn (Q.W.); 2 Key Laboratory for Ferrous Metallurgy and Resources Utilization of Ministry of Education, Wuhan University of Science and Technology, Wuhan 430081, China; 3 Hubei Provincial Key Laboratory of New Processes of Ironmaking and Steelmaking, Wuhan University of Science and Technology, Wuhan 430081, China

**Keywords:** Metallurgical waste, Copper slag, Stainless steel slag, Black ceramic tile

## Abstract

Copper slag and stainless steel slag of Electric Arc Furnace (EAF) are two typical metallurgical solid wastes, which contain a large number of valuables, such as Fe, Cr, and Cu. The transition metal elements in the waste slags, such as Cr and Fe, can be recycled as the coloring ions in the black ceramic tile. In this study, the Fe/Cr molar ratio in the raw materials of copper slag and stainless steel slag was adjusted, and the black ceramic tile was subsequently prepared by sintering. The results show that the optimum process parameters for the preparation of black ceramic tiles are the Fe/Cr molar ratio of 2.0, the sintering temperature of 1150 °C, and the sintering time of 30 min. The compressive strength of the black ceramic tile at optimum sintering conditions exceeds the minimum compressive strength of the Chinese national standard for standard polished tiles, and the concentrations of harmful elements, for example, Cr, Cu, Ni, As, Zn, Pb, and Cr(VI) are within the regulation thresholds specified by the Chinese national standard.

## 1. Introduction

According to the U.S. Geological Survey (USGS), the total global mine production of copper in 2018 was 21,000 thousand metric tons [1]. The International Copper Study Group (ICSG) predicted the growth of 2.8% in 2019 and 1.2% in 2020 as for global refined copper production [2], and China will continue to be the world’s top contributor to world refined copper growth in 2020 [3].

Copper slag is an industrial by-product of copper smelting and refining from copper ores [4]. Typically, approximately 2.2–3.0 tons of copper slag can be obtained by producing one ton of copper [5], and it is estimated that ~24.6 million tons of copper slag is produced annually [6]. There are 30–50 wt% Fe, 0.5–2.1 wt% Cu, 25–35 wt% SiO_2_ and ~1 wt% Zn in the copper slag [7,8,9,10]. Therefore, the recovery of valuable components in copper slag is of great significance.

In recent years, the output of the stainless steel of Electric Arc Furnace (EAF) has also increased continuously. According to the statistical reports of the International Stainless Steel Forum (ISSF), the global stainless steel melts shop production increased 5.5 % amount to 50.7 million tons in 2018, of which the Chinese stainless steel melt shop contributed about 52.7 % [11].

The stainless steel slag of EAF is a by-product of the stainless steel smelting process in EAF [12]. Generally, the stainless steel slag of EAF contains 40–50 % CaO, 5–10 % Fe_2_O_3_, <10 % Cr_2_O_3_, and <2 % MnO [13,14,15,16,17,18], which indicates that it has potential recovery value. However, stainless steel slag also consists of lots of hazardous elements, such as Cr, Pb, Ni, and Cd, which are harmful to the health of humans and the environment [19]. Therefore, the comprehensive utilization of stainless steel slag of EAF is of great significance to the economy and environment.

Black ceramic tiles are widely used in the building and decoration industries. In the traditional production process of black ceramic tile, the price of black ceramic tiles is closely connected with the price of Co_2_O_3_, which limits the development of cobalt-containing black ceramic products. However, by selecting the appropriate process parameters, Co-free black ceramic tiles with good performance can be prepared with industrial by-products, such as steel slag and dust, vanadium tailing, and copper slag. It can not only reduce production costs and improve economic efficiency, but also reduce environmental pollution caused by industrial wastes, which provides a new way for industrial waste recycling [20,21,22,23].

Pigments can be prepared from brick clay and metallurgical dust, and then the pigment was applied to ceramic tiles [24]. Mixtures with engobe were fired at 900 °C, whereas ceramic tiles with pigment were fired at 1060 °C. The pigment was well combined with the ceramic surface. Meanwhile, the variation of proportion of pigment to the ceramic body influence the final coloration performance of ceramic tiles.

Vanadium tailings and leather sludge can be used to prepare the black ceramic pigment [25]. The pigments based on the (Fe_0.6_Cr_0.4_)_2_O_3_ were prepared by a common solid-state reaction method. The optimum process parameters are the Fe/Cr molar ratio of 2.0, the sintering temperature of 1200°C and 40 wt% vanadium tailing. The coloring properties of prepared ceramic pigments with vanadium tailing were similar to the commercial black pigments.

Moreover, hematite tailings can be combined with quartz sand and kaolin to produce black ceramic tiles [26,27]. The sintering temperature and tailings ratio have significant effects on the properties of black ceramic tiles. The optimum process parameters for the preparation of black ceramic tiles are the addition of 55–65 wt% hematite tailings, 25 wt% kaolin, and 10–20 wt% quartz sand, the sintering temperature of 1200 °C, and the sintering time of 30 min. The properties of the prepared ceramic tiles reach the Chinese standard specifications (GB/T4100-2006) of ceramic tiles.

Steel slag can also be used to prepare for ceramic tiles [28]. The mixture of 60% clay, quartz, feldspar, talc, and 40% of steel slag can be sintered at 1200–1220 °C. The ceramic tile samples at optimum sintering conditions have good properties, with the flexural strength of 143 MPa, water absorption of 0.02 %, and linear shrinkage of 8.8 %.

According to the coloring mechanism of Fe-Cr spinel black pigment, Zhang et al. [29] added reagent grade Cr_2_O_3_ into the stainless steel dust and adjusted the Fe/Cr molar ratio in the materials. Thereafter, it was used to prepare black pigment. Ceramic tiles were prepared by adding the obtained black pigment. The optimum process parameters for the preparation of black ceramic tiles are the addition of 8 wt% black pigment, the sintering temperature of 1200 °C, and the sintering time of 30 min. The compressive strength of the black ceramic tiles and the concentrations of harmful elements are within the regulation thresholds specified by the Chinese national standard.

Generally, the traditional technology of preparing black ceramic tile is a two-step process, i.e., black pigment preparation stage and ceramic tiles preparation stage. Note that Fe- and Cr-containing black ceramic pigments are the most extensively used pigment in porcelain tile production. SiO_2_ and Al_2_O_3_ are the major components of the ceramic matrix [20]. Copper slag and EAF stainless steel slag are rich in oxides of SiO_2_, Al_2_O_3_, Fe_2_O_3_, and Cr_2_O_3_, and can be potentially prepared black ceramic tiles directly. This can not only reduce energy consumption and production costs, but also reduce the environmental hazards of heavy metals in the slag and recycle the valuable metals in the slag.

In this study, copper slag and stainless steel slag of EAF were characterized by ICP-AES, SEM, TG/DTA, and XRD. Then, the Fe-Cr-based black ceramic tiles were prepared with copper slag and stainless steel slag by adjusting the Fe/Cr molar ratio in the mixture. The effects of the process parameters such as Fe/Cr molar ratio, sintering temperature and sintering time on the microstructure, phase composition, coloration performance and compressive strength of the ceramic tile were studied in order to provide theoretical and experimental basis for the harmless and value-added application of copper slag and stainless steel slag of EAF.

## 2. Experimental

### 2.1. Raw Materials

The waste copper slag was collected from a copper smelting plant in central China where the copper slag was impoverished and floated for copper recovery. The stainless steel slag of EAF was sampled in a domestic stainless steel mill.

### 2.2. Characterization

#### 2.2.1. Chemical Composition, Crystalline Phase, and Microstructure

The chemical compositions of copper slag and stainless steel slag were analyzed by inductively coupled plasma emission spectrometer (ThemoElemental IRIS Advantage Radial, America). The crystalline phases were identified using an X-ray powder diffractometer (PANalytical X’Pert PRO MPD, Netherlands) with Cu Kα radiation at a tube voltage of 40 kV, current of 40 mA and 2 theta scanning ranging from 15° to 80°. The microstructures of the waste slags were examined with a field emission scanning electron microscope (FEI Nova NanoSEM400, FEI, Hillsboro, Oregon, America).

#### 2.2.2. Thermogravimetric Test

Approximately 10 mg copper slag and 10 mg stainless steel slag were, respectively, weighed and heated to 1200 °C at the heating speed of 10 °C /min in the air atmosphere using a thermogravimetric analyzer (NETZSCH STA 449F3, Selb, Germany) to monitor the relationship between the gravity and temperature of copper slag and EAF stainless steel slag.

### 2.3. Preparation of Black Ceramic Tile

The copper slag and the EAF stainless steel slag were ground to a particle size of 200 mesh or less using a planetary ball mill (QM-3SP4, Nanjing Chi Shun Technology Development Co., Ltd, Nanjing, China) and dried in an oven at 100 °C for 24 hours. It was subsequently mixed in a certain ratio (Fe/Cr molar ratio is 0.5–2.5), and pressed into cylindrical specimens with a diameter of ~2 cm using a conventional press at a pressure of 25 MPa. Then, the samples were placed in a muffle furnace (SX2-10-13, Shanghai Shi Yan Electric Furnace Co., Ltd, Shanghai, China) and sintered in an air atmosphere at ambient pressure to obtain black ceramic tile samples. The experimental process parameters are shown in Table 1. The heating speed of the furnace was about 10 °C/min and the samples were cooled down to room temperature in the muffle furnace. Subsequently, the long-term leaching behavior and physical properties of ceramic cylinders were tested.

### 2.4. Coloration Performance of Black Ceramic Tiles

The L*, a*, and b* color space developed by the International Commission on Illumination (CIE) is a device-independent color model. In this system, L* is the degree of lightness and darkness of the color in relation to the scale extending from white (L* = 100) to black (L* = 0). a* is the degree of green (−a*) and red (+a*), b* is the degree of blue (−b*) and yellow (+b*) [25]. In this work, the color of the ceramic tile was characterized by L*, a*, and b* color space, and the L*, a*, and b* values of the tile were measured using a portable chromatic aberration meter (SC10 NR10QC). For black ceramic titles, the closer L*, a*, and b* are to 0, the better the coloration performance.

### 2.5. Leaching Test and Compression Resistance of Black Ceramic Tiles

In this study, the environmental protection industry standard of China HJ/T299-2007 [30] was adopted to carry out the leaching test of toxic substances in ceramic tiles. In this test, concentrated nitric acid and concentrated sulfuric acid with the mass ratio of 1:2 were firstly added to deionized water to prepare the leaching agent with the pH of 3.2 ± 0.05. The powder sample and the leaching agent were added to a 100 ml glass bottle and sealed to ensure the solid and liquid ratio of 1:10 (g/mL). At 23 ± 2 °C, the mixture was shaken by an end-over-end homogenizer with the rotation speed of 30 ± 2 r/min for 18 ± 2 h. After filtration, the contents of Cr, Cu, Ni, As, Zn, Pb, and Cr(VI) in the leachate were measured. The concentration of Cr(VI) was determined by spectrophotometry (Model 722 spectrophotometer, Beijing Century Science Instrument Co., Ltd, Beijing, China), whereas the concentration of other leachable toxic elements was determined by the ICP-AES. The ceramic tile was also subjected to a compression test using a hydraulic universal test machine (WE-30, Tai Tian machinery Jiangsu Co., Ltd, Jiangsu, China).

## 3. Results and Discussion

### 3.1. Characterization of Copper Slag and Stainless Steel Slag

#### 3.1.1. Chemical Composition and Crystalline Phases

The chemical compositions of EAF stainless steel slag and copper slag are shown in Table 2. The main chemical constituents of copper slag used in this study are Fe_2_O_3_ and SiO_2_, the content of them are 52.69% and 33.44%, respectively, whereas the main chemical constituents of stainless steel slag are SiO_2_, CaO, and Cr_2_O_3_, which account for about 70 % of the total content.

**Figure 1:**
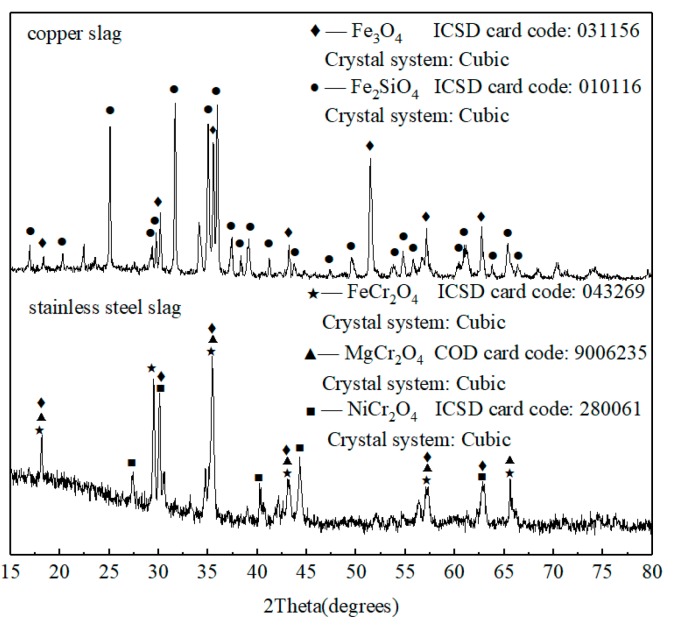
X-ray diffraction (XRD) patterns of waste copper slag and stainless steel slag.

Figure 1 shows the XRD patterns of copper slag and stainless steel slag of EAF, respectively. It indicates that the main crystalline phases of copper slag are Fe_2_SiO_4_ and Fe_3_O_4_. The stainless steel slag mainly consists of spinels, such as FeCr_2_O_4_, MgCr_2_O_4_, NiCr_2_O_4_, and Fe_3_O_4_, which is consistent with previous studies [7,9,13,14,15].

#### 3.1.2. Thermogravimetric Analysis

**Figure 2:**
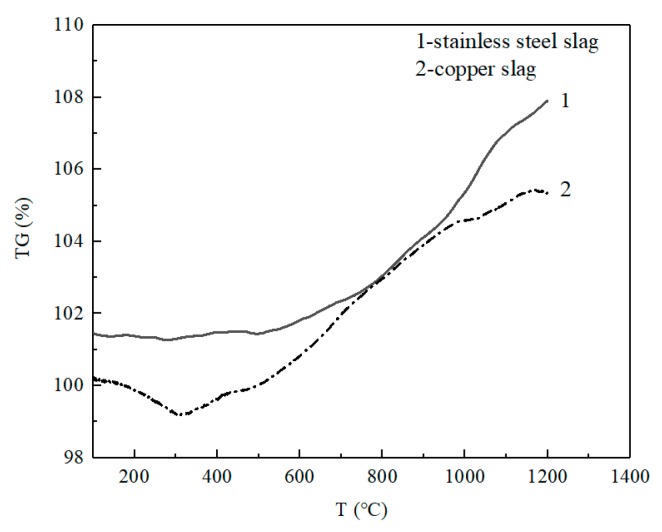
Thermogravimetric curves of copper slag and stainless steel slag of electric arc furnace (EAF).

Thermogravimetric curves of copper slag and stainless steel slag are shown in Figure 2. The results show that the mass of copper slag decreases with increasing temperature below 310 °C, mainly due to the presence of moisture in the slag and the dehydration reaction of the crystalline hydrate in the copper slag. The mass of the copper slag increases with the temperature above 310 °C, which is mainly due to the oxidation of Fe_2_SiO_4_ and Fe_3_O_4_ in the slag and the chemical reaction are as shown in the Equations (1) and (2) [6,7,9]. The mass of the stainless steel slag of EAF increases with temperature after 500 °C possibly due to the oxidation of Fe_3_O_4_ in the slag.

2Fe_2_SiO_4_ + O_2_→2Fe_2_O_3_ + 2SiO_2_     Δ*G^θ^* = −501646 + 201.7*T* (J/mol) (1)

4Fe_3_O_4_ + O_2_→6Fe_2_O_3_      Δ*G^θ^* = −477658 + 277.2*T* (J/mol) (2)

### 3.2. Effect of Fe/Cr Molar Ratio on the Coloration Performance of Ceramic Tiles

**Figure 3:**
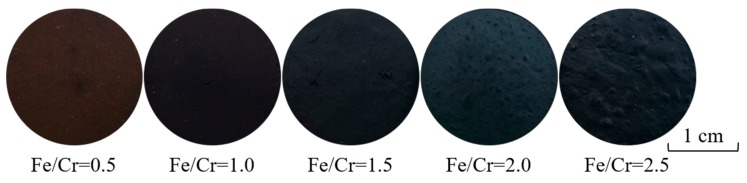
Ceramic tile samples with different Fe/Cr molar ratio sintered at 1150 °C for 30 min.

**Figure 4:**
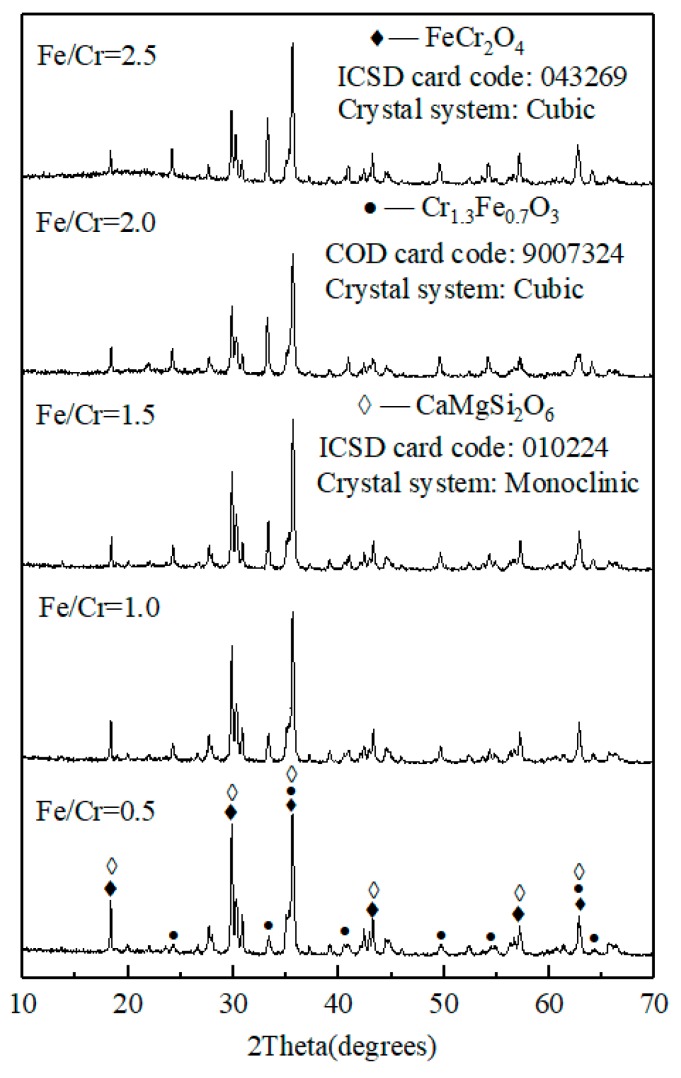
XRD patterns of ceramic tiles with different Fe/Cr molar ratio sintered at 1150 °C for 30 min.

Figure 3 and Figure 4 show the photograph of the ceramic tile samples sintered at different Fe/Cr molar ratios and the XRD patterns of ceramic tiles prepared under different Fe/Cr molar ratios, respectively. It can be found in Figure 3 that the color of ceramic tiles become darker as the Fe/Cr molar ratios increased from 0.5 to 2.0. Moreover, there are no cracks or defects on the surface of ceramic tiles. However, when the Fe/Cr =2.5, the tile surface is slightly expanded. As shown in Figure 4, the chromite spinel (FeCr_2_O_4_) is the main crystalline phase of black ceramic tile samples prepared at 1150 °C. Moreover, Cr_1.3_Fe_0.7_O_3_ also present in the ceramic tile samples, which is formed by the reaction of Fe_2_O_3_ and Cr_2_O_3_. Meanwhile, CaMgSi_2_O_6_ exists as a minor component. With the increase of Fe/Cr molar ratio, the contents of Cr_1.3_Fe_0.7_O_3_ and FeCr_2_O_4_ increase, whereas the content of Fe_2_O_3_ decreases, possibly due to the further reaction of Fe_2_O_3_ and Cr_2_O_3_ in the mixture to form Cr_1.3_Fe_0.7_O_3_ and FeCr_2_O_4_.

**Figure 5:**
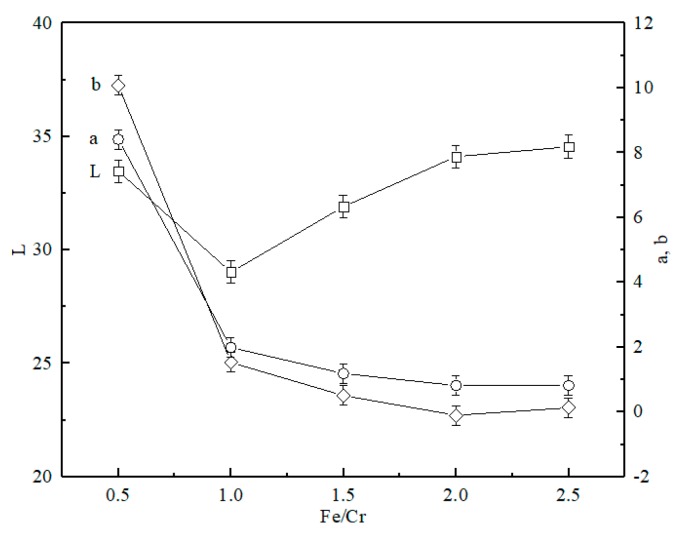
Effects of Fe/Cr molar ratio on the coloration of ceramic tiles sintered at 1150 °C for 30 min.

Figure 5 shows the effects of the Fe/Cr molar ratio on the coloration of ceramic tiles. As the Fe/Cr molar ratio increases, the L* value decreases firstly. This is because the increased Fe_2_O_3_ content in the material reacts with Cr_2_O_3_ to generate large amounts of Cr_1.3_Fe_0.7_O_3_ and FeCr_2_O_4_. Therefore, it enhances the black chromaticity value of the ceramic tile. Moreover, note that when the Fe/Cr molar ratio is higher than 1.0, L* value increases gradually. This is possibly due to that the increased Fe_2_O_3_ and Cr_2_O_3_ reaction at this time mainly generate Cr_1.3_Fe_0.7_O_3_, while FeCr_2_O_4_ content is relatively less than that in Fe/Cr = 1.0, and weakens the black chromaticity value of the ceramic tile.

As the Fe/Cr molar ratio increase in the materials, the values of +a* and +b* gradually decrease. The ceramic tile sample becomes reddish with the increasing of +a* value. This may be due to the presence of small amounts of unreacted Fe_2_O_3_ in the ceramic tile. A larger value of +b* indicates that the sample is yellowish and certain amounts of Ca are contained in ceramic tiles, which may influence the coloration of Fe^3+^ to make it brownish [29].

Therefore, the optimum Fe/Cr molar ratio of the mixed slag material for better coloration performance is 2.0, after considering the changes in the values of L*, a*, and b* of black ceramic tile and the contents of Cr_1.3_Fe_0.7_O_3_ and FeCr_2_O_4_.

### 3.3. Effect of Sintering Temperature on the Coloration Performance of Ceramic Tiles

**Figure 6:**
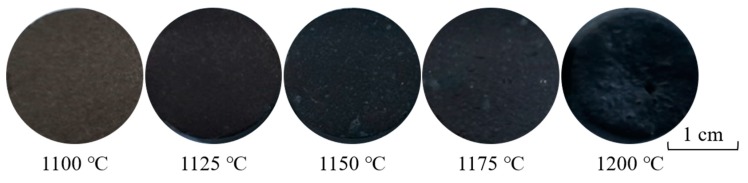
Ceramic tile samples with Fe/Cr = 2.0 sintered at different temperatures for 30 min.

**Figure 7:**
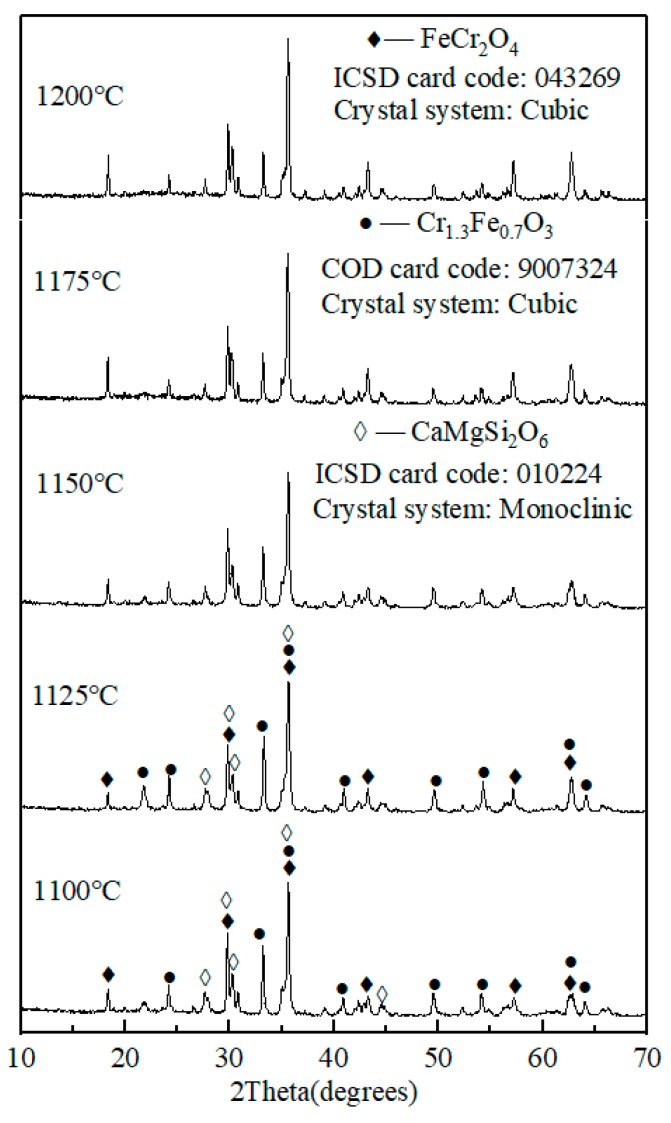
XRD patterns of ceramic tiles with Fe/Cr =2.0 sintered at different temperatures for 30 min.

Figure 6 and Figure 7 show the photograph of ceramic tile samples sintered at different sintering temperatures and the XRD patterns of ceramic tiles prepared under different sintering temperatures, respectively. It was found in Figure 6 that as the sintering temperature increases from 1100 °C to 1150 °C, the color of ceramic tile becomes become darker. The ceramic tile is black and smooth after being sintered at 1125 °C, 1150 °C, and 1175 °C. However, when the sintering temperature increases to 1200 °C, the ceramic tiles become bulging deformation. As shown in Figure 7, the main phases of ceramic tiles are FeCr_2_O_4_, CaMgSi_2_O_6_, and Cr_1.3_Fe_0.7_O_3_. As the sintering temperature increases, the contents of Cr_1.3_Fe_0.7_O_3_ and CaMgSi_2_O_6_ firstly increase and then decrease, while the FeCr_2_O_4_ firstly decreases and then increases. Meanwhile, when the sintering temperature rises to 1200 °C or even higher, more liquid phases will be formed in the ceramic tile and could dramatically lead to the dissolution of the Cr_1.3_Fe_0.7_O_3_ [29], which is not conducive to ceramic tile production.

**Figure 8:**
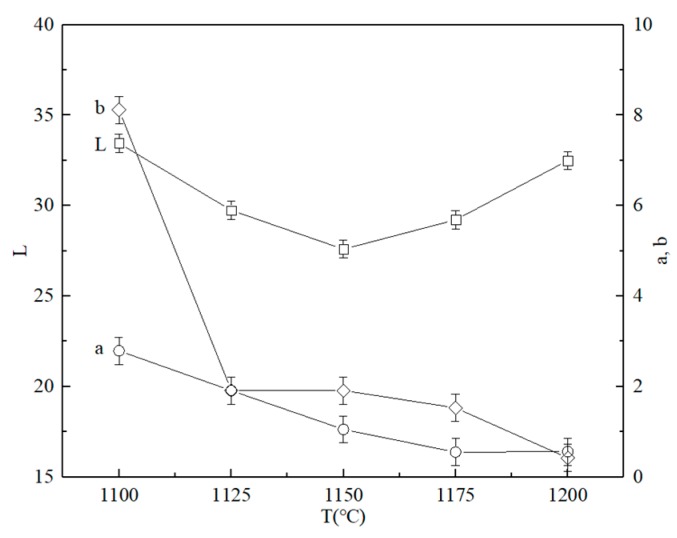
Effects of sintering temperature on the coloration of ceramic tiles with Fe/Cr =2.0 sintered for 30 min.

Figure 8 shows the effects of sintering temperature on the coloration performance of ceramic tiles. As the sintering temperature increases, the L* value decreases firstly and then increases. At 1150 °C, the L* value of ceramic tile is the smallest. With the increase of sintering temperature, the values of a* and b* decrease. Moreover, the value of a* attains the minimum at 1175 °C and keeps stable, and when the sintering temperature is higher than 1125 °C, the b* value remains low with the further increase of sintering temperature. It is mainly due to the fact that the increase of temperature increases the chemical reaction ability and diffusion ability of solid particles, the Fe_2_O_3_, and Cr_2_O_3_ in the mixture react to form a large amount of FeCr_2_O_4_ and Cr_1.3_Fe_0.7_O_3_, which enhances the black chromaticity value of the ceramic tile. In addition, as the sintering temperature further increases, the reaction of Fe_2_O_3_ and Cr_2_O_3_ in the mixture mainly generates FeCr_2_O_4_, while the content of Cr_1.3_Fe_0.7_O_3_ is relatively low, which weakens the black chromaticity value of the black ceramic tile.

**Figure 9:**
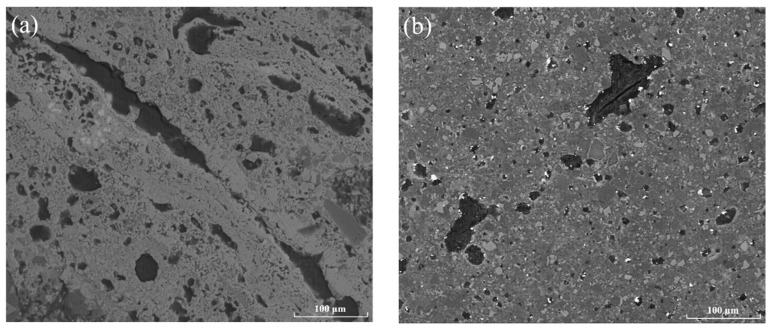
SEM images of ceramic tiles with Fe/Cr = 2.0 sintered at different temperatures for 30 min (**a**) 1100 °C and (**b**) 1150 °C.

Figure 9 shows the microstructure of black ceramic tiles sintered at 1100 °C and 1150 °C. It can be seen from Figure 9 that there are a lot of micropores in the ceramic tile fired at 1100 °C. When fired at 1150 °C, the number of micropores decreases and the oxide phases of iron and chromium are evenly distributed. The results show that increasing sintering temperature can promote the sintering reaction of the black ceramic tile, in particular, an appropriate amount of liquid phase can be generated to reduce the porosity, thereby improving the surface quality and coloration performance of the black ceramic tile.

Therefore, the optimum sintering temperature is 1150 °C, after considering the changes in the values of L*, a*, and b*, the contents of Cr_1.3_Fe_0.7_O_3_ and FeCr_2_O_4_ and the microstructure of black ceramic tile.

### 3.4. Effects of Sintering Time on the Coloration Performance of Ceramic Tiles

**Figure 10:**
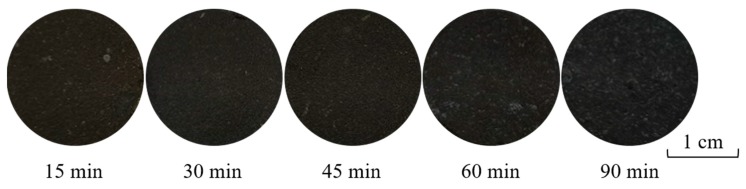
Ceramic tile samples with Fe/Cr = 2.0 sintered at 1150 °C for different time.

**Figure 11:**
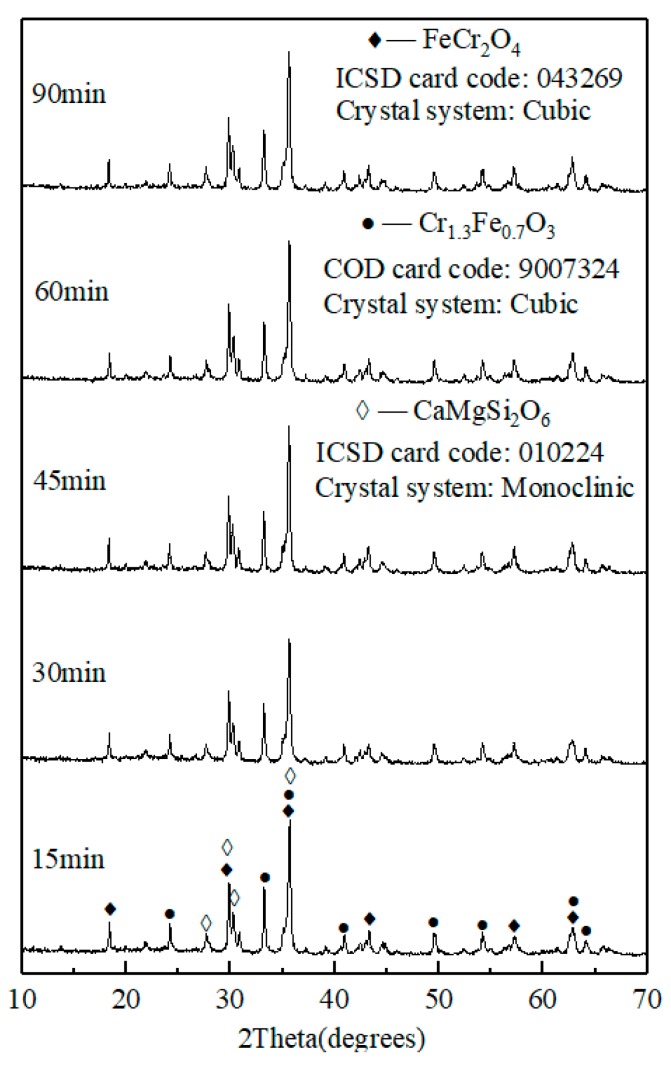
XRD patterns of ceramic tiles with Fe/Cr = 2.0 sintered at 1150 °C for different time.

Figure 10 and Figure 11 show the photograph of ceramic tile samples sintered at different sintering time and the XRD diagrams of black ceramic tiles prepared under different sintering times, respectively. It was found in Figure 10 that the color of ceramic tiles is dark as the sintering temperature increases from 15 min to 90 min. The surface of ceramic tiles is smooth and black with uniform coloration. As shown in Figure 10, at the sintering temperature of 1150 °C for sintering time ranged from 15 to 90 min, the main phases of ceramic tile are FeCr_2_O_4_, CaMgSi_2_O_6_ and Cr_1.3_Fe_0.7_O_3_. The results show that under the sintering temperature of 1150 °C, the chemical reaction of the ceramic tile reacts completely within a short time.

**Figure 12:**
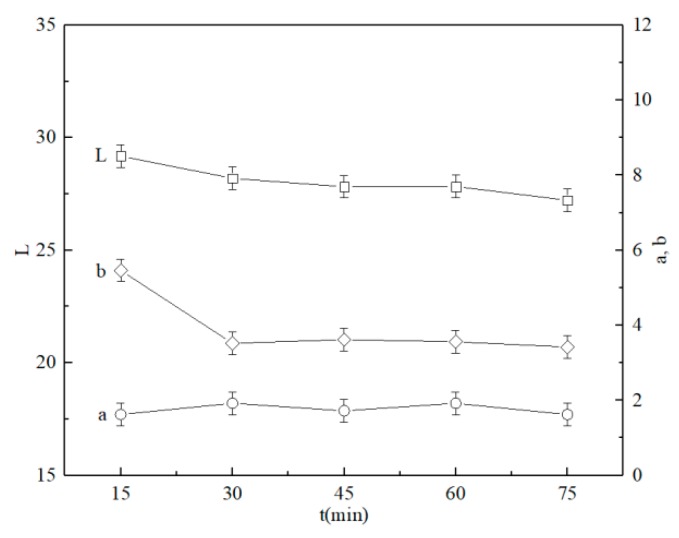
Effects of sintering time on the coloration of ceramic tiles with Fe/Cr = 2.0 sintered at 1150 °C.

The effects of sintering time on the ceramic tile color are related to the reaction of the coloring ions with the ceramic matrix. Figure 12 shows the effects of sintering time on the coloration performance of black ceramic tiles. As shown in Figure 12, when the sintering temperature is 1150 °C, the L*, a*, and b* of the black ceramic tile generally decrease with the increase of the sintering time. The b* of the black ceramic tile is slightly increased, i.e., the tile surface is yellowish-brown, possibly due to the oxidation of iron oxides [31].

Therefore, the optimum sintering time is 30 min, after considering the changes in the values of L*, a* and b* and the phase compositions of black ceramic tile.

### 3.5. Compression and Leaching Tests of Ceramic Tiles

Table 3 shows the compression test results of black ceramic tiles. The results show that as the Fe/Cr increases, the compressive strength of the black ceramic tile increases gradually sintering at 1150 °C for 30 min. At the sintering temperature range from 1100 °C to 1200 °C, the compressive strength increases firstly and then decreases. This is due to the fact that when the sintering temperature rises to 1200 °C or even higher, it can lead to overburning of the ceramic tile, resulting in cracks on the ceramic tile surface and reduction of compressive strength. In addition, it attains a maximum value of 50.58 MPa at 1150 °C. Moreover, as the sintering time increases, the compressive strength of black ceramic tiles decreases. Note that when the Fe/Cr molar ratio of 2.0, sintering temperature of 1150 °C, and sintering time of 30 min, the compressive strength of black ceramic tile is 50.58 MPa, which exceeds the minimum compressive strength of 27 MPa of the Chinese national standard (GB/T4100-2006) [32] for standard polished tiles.

The leaching toxicity results of the ceramic tile with Fe/Cr = 2.0 sintered at 1150 °C for 30 min are shown in Table 4. The results show that the concentrations of harmful elements such as Cr, As, Zn, Pb, and Cr(VI), can meet the regulation thresholds specified by the national standard (GB 5085.3-2007) [33]. Therefore, the use of copper slag and stainless steel slag of EAF to prepare black ceramic tile is a safe and value-added process that can harmlessly recycle waste slag.

## 4. Conclusions

This study provides a new way to develop low-cost, high-quality, environmentally friendly black ceramic tile, and achieves the harmless and value-added utilization of copper slag and stainless steel slag of EAF. The black ceramic tiles can be used in the building and decoration industries. Its application prospect is relatively broad.

(1) The optimum process parameters for the preparation of black ceramic tile from copper slag and EAF stainless steel slag are Fe/Cr = 2.0, the sintering temperature of 1150 °C, and sintering time of 30 min.

(2) The main crystalline phases of the black ceramic tile prepared under the optimum process parameters are chromite spinel (FeCr_2_O_4_) and solid solution oxide (Cr_1.3_Fe_0.7_O_3_), with fewer micropores and more uniform distribution of iron and chromium oxide phase.

(3) Under the optimum technological parameters, the surface of ceramic tiles is smooth and black with uniform coloration (L* = +28.19, a* = +1.71 and b*= +3.52). The compressive strength of the black ceramic tiles exceeds the minimum compressive strength of the Chinese national standard for standard polished tiles, and the concentrations of harmful elements such as Cr, Cu, Ni, As, Zn, Pb, and Cr(VI) are within the regulation thresholds specified by the national standard.

## Figures and Tables

**Table 1:** The process parameters of black ceramic tile production.

Fe/Cr Molar Ratio	Sintering Temperature (°C)	Sintering Time (min)
0.5, 1.0, 1.5, 2.0, 2.5	1100, 1125, 1150, 1175, 1200	15, 30, 45, 60, 90

**Table 2:** The chemical components of copper slag and stainless steel slag (wt%).

Samples	SiO_2_	Fe_2_O_3_	Al_2_O_3_	CaO	MgO	Cr_2_O_3_	CuO	ZnO	NiO
Copper slag	33.44	52.69	5.68	2.90	2.03	0.24	0.24	2.01	-
Steel slag	38.61	7.32	9.52	16.03	8.07	14.57	0.03	0.01	0.71

**Table 3:** Compressive strength of ceramic tile at different sintering conditions (MPa).

No.	Sintering Conditions	Compressive Strength
1	Fe/Cr = 1.0 1150 °C 30min	21.71
2	Fe/Cr = 1.5 1150 °C 30 min	30.09
3	Fe/Cr = 2.0 1150 °C 15 min	58.12
4	Fe/Cr = 2.0 1150 °C 30 min	50.58
5	Fe/Cr = 2.0 1100 °C 30 min	21.36
6	Fe/Cr = 2.0 1200 °C 30 min	8.23
7	Fe/Cr = 2.0 1150 °C 45 min	23.68

**Table 4:** Concentrations of leachable elements from tiles sintered with Fe/Cr = 2.0 at 1150 °C and 30 min (mg/L).

Leachable Elements	Cr	Cu	Ni	As	Zn	Pb	Cr(VI)
Ceramic tile samples	2.35	0.45	0.14	0.082	1.59	0.19	0.024
GB 5085.3-2007	15	100	5	5	100	5	5

## References

[1] Mineral Commodity Summaries Copper. https://prd-wret.s3-us-west-2.amazonaws.com/assets/palladium/production/s3fs-public/atoms/files/mcs-2019-coppe.pdf.

[2] ICSG Raises 2019 Copper Deficit Prediction. https://investingnews.com/daily/resource-investing/base-metals-investing/copper-investing/icsg-raises-copper-deficit-prediction/.

[3] ICSG Press Release: Copper Market Forecast 2019/2020. https://recyclingportal.eu/Archive/48147.

[4] Rajasekar A., Arunachalam K., Kottaisamy M. (2019). Assessment of strength and durability characteristics of copper slag incorporated ultra high strength concrete. J. Cleaner Prod..

[5] Prem P.R., Verma M., Ambily P.S. (2018). Sustainable cleaner production of concrete with high volume copper slag. J. Cleaner Prod..

[6] Gorai B., Jana R.K., Premchand (2003). Characteristics and utilisation of copper slag-a review. Resour., Conserv. Recycl..

[7] Fan Y., Shibata E., Iizuka A., Nakamura T. (2014). Crystallization behaviors of copper smelter slag studied using time-temperature-transformation diagram. Mater. Trans..

[8] Feng Y., Kero J., Yang Q., Chen Q., Engström F., Samuelsson C., Qi C. (2019). Mechanical activation of granulated copper slag and its influence on hydration heat and compressive strength of blended cement. Materials.

[9] Fan Y., Shibata E., Iizuka A., Nakamura T. (2015). Crystallization behavior of copper smelter slag during molten oxidation. Metall. Mater. Trans. B.

[10] Holland K., Eriç R.H., Taskinen P., Jokilaakso A. (2019). Upgrading copper slag cleaning tailings for re-use. Miner. Eng..

[11] Stainless Steel Production Reached 50.7 Million Metric Tons in 2018. http://www.worldstainless.org/news/show/2351.

[12] Li J., Mou Q., Zeng Q., Yu Y. (2019). Experimental study on precipitation behavior of spinels in stainless steel-making slag under heating treatment. Processes.

[13] Fathy S., Guo L., Ma R., Gu C., Sun W. (2018). Properties of steel slag and stainless steel slag as cement replacement materials: A comparative study. J. Wuhan Univ. Technol., Mater. Sci. Ed..

[14] Wu T., Zhang Y., Zhao Z., Yuan F. (2019). Effects of Fe_2_O_3_ on reduction process of Cr-containing solid waste self-reduction briquette and relevant mechanism. Metals.

[15] Fan W., Yang Q., Guo B., Zhang S. (2018). Crystallization mechanism of glass-ceramics prepared from stainless steel slag. Rare Met..

[16] Zeng Q., Li J., Mou Q., Zhu H., Xue Z. (2019). Effect of FeO on spinel crystallization and chromium stability in stainless steel-making slag. JOM..

[17] Galan-Arboledas R.J., Alvarez de Diego J., Dondi M., Bueno S. (2017). Energy, environmental and technical assessment for the incorporation of EAF stainless steel slag in ceramic building materials. J. Cleaner Prod..

[18] Davydenko A., Karasev A., Glaser B., Jönsson P. (2019). Direct reduction of Fe, Ni and Cr from oxides of waste products used in briquettes for slag foaming in EAF. Materials.

[19] Zhang H., Hong X. (2011). An overview for the utilization of wastes from stainless steel industries. Resour. Conserv. Recycl..

[20] Zhu R., Ma G., Cai Y., Chen Y., Yang T., Duan B., Xue Z. (2016). Ceramic tiles with black pigment made from stainless steel plant dust: Physical properties and long-term leaching behavior of heavy metals. J. Air Waste Manage. Assoc..

[21] Llusar M., Bermejo T., Primo J.E., Gargori C., Esteve V., Monrós G. (2017). Karrooite green pigments doped with Co and Zn: Synthesis, color properties and stability in ceramic glazes. Ceram. Int..

[22] Dondi M., Zanelli C., Ardit M., Cruciani G., Mantovani L., Tribaudino M., Andreozzi G.B. (2013). Ni-free, black ceramic pigments based on Co-C-Fe-Mn spinels: A reappraisal of crystal structure, colour and technological behavior. Ceram. Int..

[23] Carneiro J., Tobaldi D.M., Capela M.N., Seabra M.P., Labrincha J.A. (2019). Waste-Based pigments for application in ceramic glazes and stoneware bodies. Materials.

[24] Ovčačíková H., Vlček J., Klárová M., Topinková M. (2017). Metallurgy dusts as a pigment for glazes and engobes. Ceram. Int..

[25] Du M., Du Y., Chen Z., Li Z., Yang K., Lv X., Feng Y. (2017). Synthesis and characterization of black ceramic pigments by recycling of two hazardous wastes. Appl. Phys. A: Mater. Sci. Process..

[26] Chen Y., Zhang Y., Chen T., Liu T., Huang J. (2013). Preparation and characterization of red porcelain tiles with hematite tailings. Constr. Build. Mater..

[27] Tanisana B., Turanb S. (2011). Black ceramic pigments for porcelain tile bodies produced with chromite ores and iron oxide waste. J. Ceram. Process. Res..

[28] Zhao L., Li Y., Zhou Y., Cang D. (2014). Preparation of novel ceramics with high CaO content from steel slag. Mater. Des..

[29] Zhang X., Ma G., Jin Y., Cheng P. (2014). Preparation of ceramic tiles with black pigments using stainless steel plant dust as a raw material. Ceram. Int..

[30] (2007). Solid waste-Extraction procedure for leaching toxicity-Sulphuric acid & nitric acid method.

[31] Corradi A.B., Leonelli C., Manfredini T., Pozzo P., Romagnoli M. (1993). Preparation and properties of fast-fired porcelain tiles containing natural Chromite. Am. Ceram. Soc. Bull..

[32] (2006). Ceramic Tiles.

[33] (2007). Identification Standards for Hazardous Wastes-Identification for Extraction Toxicity.

